# Disentangling the Contribution of Spatial Reference Frames to Executive Functioning in Healthy and Pathological Aging: An Experimental Study with Virtual Reality

**DOI:** 10.3390/s18061783

**Published:** 2018-06-01

**Authors:** Silvia Serino, Francesca Morganti, Desirée Colombo, Elisa Pedroli, Pietro Cipresso, Giuseppe Riva

**Affiliations:** 1 Applied Technology for Neuro-Psychology Lab, IRCCS Istituto Auxologico Italiano, Via Magnasco 2, 20149 Milan, Italy; e.pedroli@auxologico.it (E.P.); pietro.cipresso@unicatt.it (P.C.); giuseppe.riva@unicatt.it (G.R.); 2 Department of Psychology, Università Cattolica del Sacro Cuore, Largo Gemelli 1, 20100 Milan, Italy; 3 Department of Human and Social Sciences, University of Bergamo, Piazzale S. Agostino 2, 24129 Bergamo, Italy; francesca.morganti@unibg.it; 4 Department of Basic Psychology, Clinic and Psychobiology, Universitat Jaume I, Av. Sos Baynat, s/n, 12071 Castellón, Spain; dcolombo@uji.es

**Keywords:** virtual reality, allocentric abilities, executive functions, syncing abilities, neurodegenerative diseases

## Abstract

A growing body of evidence pointed out that a decline in effectively using spatial reference frames for categorizing information occurs both in normal and pathological aging. Moreover, it is also known that executive deficits primarily characterize the cognitive profile of older individuals. Acknowledging this literature, the current study was aimed to specifically disentangle the contribution of the cognitive abilities related to the use of spatial reference frames to executive functioning in both healthy and pathological aging. 48 healthy elderly individuals and 52 elderly suffering from probable Alzheimer’s Disease (AD) took part in the study. We exploited the potentiality of Virtual Reality to specifically measure the abilities in retrieving and syncing between different spatial reference frames, and then we administrated different neuropsychological tests for evaluating executive functions. Our results indicated that allocentric functions contributed significantly to the planning abilities, while syncing abilities influenced the attentional ones. The findings were discussed in terms of previous literature exploring relationships between cognitive deficits in the first phase of AD.

## 1. Introduction

To refer to the position of an object in our environment, for example, a bottle on the table, we may simply use the term “left” or “right”, adopting therefore our body as reference for its spatial location (“The blue bottle is on my right”). However, if our spatial position changes, the position of the bottle “changes” accordingly. To maintain the spatial position of the bottle in “mind”, we have to refer to the relationship existing with other elements in the surroundings, for example the table itself (“The blue bottle is on the table”). These are the two fundamental types of coordinate used to identify locations in space: the egocentric and allocentric spatial reference frames [1,2]. They are also used to refer to spatial entities in language (for a review, see [3]), thus implying that these frames are two crucial modes for organizing information in our entire cognitive system (for their impact on memory, see [4]). Within the egocentric reference frame, which is defined by subject-to-object relationships (“The blue bottle is on my right”), information is categorized in relation to the self. Conversely, in an allocentric reference frame, which is formed by object-to-object relations (“The blue bottle in on the table”), information is categorized independently from the self, and it’s related to other elements in the environment [5,6]. Influential studies in developmental psychology demonstrated that children initially adopt egocentric reference frames to categorize information; only later, they become able to control both frames [7,8].

The process of encoding, storing and retrieval spatial information from environment has been modelled in cognitive neuroscience [6,9]. While moving and interacting with the environment, individuals encode information within an egocentric reference frame within the parietal lobe [6,9,10]. Then, egocentric representations are transformed into allocentric representations for long-term storage in medial temporal lobes [6,9,10] thanks to the contribution of the retrosplenial cortex (RCS) [11]. When it is necessary to retrieve an allocentrically-coded representation (for example, I have to retrieve the position of a supermarket previously visited during a tour in a city), the same transformation circuitry should be activated backwards [6,9]. Moreover, the existence of a specific process underlying this egocentric–allocentric transformation, i.e., the Mental Frame Syncing (MFS), has recently been proposed, which is supposed to be crucial to support the recall of spatial information. Indeed, the MFS operates by placing the egocentric heading into the allocentric representation, making easy the translation of this stored abstract allocentric map into an egocentric representation [12,13,14]. In the previous example, when I have to remember the position of the supermarket, I must also remember my egocentric heading with respect to the supermarket and synchronize these two kinds of information. 

Remarkably, the capacity to store allocentric representations seems to be the key spatial ability underlying the decline in navigation abilities observed in both individuals suffering from Alzheimer’s Disease (AD) and Mild Cognitive Impairment (MCI) [15,16], especially because the neurodegeneration started in the medial temporal lobe and related areas [17,18,19,20,21]. However, it is also known that a decline in spatial abilities occurs also in normal aging, involving both spatial memory [22,23,24] and spatial navigation [16]. More specifically, an increasing number of studies emphasized a decline of allocentric abilities after the 60 years of age, which was related with the physiological deterioration of hippocampal areas [25,26,27,28]. 

However, a growing body of evidence also suggested an association between the ability in using spatial reference frames for memory and navigation, and other cognitive abilities, such as executive functions [29,30,31]. Indeed, it has been suggested that the general age-related cognitive decline is particularly linked to the weakening of executive functioning [32], which comprises a set of different cognitive abilities necessary to plan, organize, execute and monitor actions. This age-related decline in executive functions can negatively affect performance in spatial navigation [33], since it implies the correct organization of actions within the environment, the monitoring of their outcome, and the search for another strategy if it fails. In this vein, an interesting study [30] employing a Virtual Reality (VR)-based navigation task indicated for older adults a reduced activation in brain areas typically involved in allocentric navigation (i.e., the hippocampus, the parahippocampal gyrus, the retrosplenial cortex and the parietal lobes), and a greater frontal lobe activation (typically associated with the executive functioning). In the same direction, more recently, Laczò and co-workers [29] demonstrated the presence of an association between allocentric navigation and executive functions in a sample of individuals with amnestic MCI.

Understanding the association between deficits, spatial reference frames, and executive impairments in healthy and pathological aging represents an opportunity to highlight which cognitive markers primarily characterize neurodegenerative diseases.

In this perspective, VR offers tremendous advantages for specifically investigating allocentric and egocentric abilities [15,34]. Besides the opportunity for an ecological, controlled and secure testing, within virtual environments, it is possible to set-up a “reorientation task” by systematically varying the starting point of the retrieval phase with respect to the encoding phase [35,36]. This strategy (i.e., the virtual disorientation) forces participants to refer to their stored allocentric map and synchronize it with new egocentric input (i.e., the MFS ability) to orient in the environment [37,38,39]. In a recent study from our group, we exploited the potential of VR to study the presence of allocentric and syncing deficits in a sample of patients with AD and amnestic MCI. We found that the cognitive profile of amnesic MCI patients was marked by an allocentric impairments. Instead, a more subtle deficit in the synchronization ability was found in patients suffering from AD.

To further investigate the association between the ability to use spatial reference frames and executive functions, in the current study, we recruited 48 healthy elderly individuals and 52 elderly individuals suffering from probable AD. Accordingly, we employed a VR-based task (adopted in a previous study [39]) to measure allocentric and syncing abilities, and then, we administrated different neuropsychological tests for evaluating executive functions. The aim was to specifically disentangle the contribution of the cognitive abilities related to the use of spatial references frames to executive functioning in both healthy and pathological aging.

## 2. Materials and Methods

### 2.1. Participants

100 elderly subjects participated in the study: 48 healthy elderly individuals and 52 elderly individuals suffering from probable AD. The healthy elderly individuals were recruited from a panel of volunteers. Inclusion criteria were: (1) age older than 65 years old; (2) no history or presence of psychiatric or neurological disorders (evaluated with a brief interview); (3) normal or corrected-to-normal vision; (4) scores on Mini-Mental State Examination—MMSE [40] over than 27. AD patients were recruited from different social senior centres located in Lombardy (Italy). Inclusion criteria for individuals with AD were: (1) age older than 65 years old; (2) diagnosis of AD according to NINCDS-ARDRA criteria made by neurologist/geriatric staff of social senior centre [41]; (3) no history or presence of psychiatric or neurological disorders other than AD; (4) normal or corrected-to-normal vision; and (5) scores on Milan Overall Dementia Scale [42] under 85.5 (i.e., clinical cut-off for probable dementia due to Alzheimer’s Disease).

AD patients had a mean score at Mini-Mental State Examination—MMSE [40] of 21.54 (SD = 2.71), while the healthy elderly individuals had a mean score of 28.86 (SD = 1.15). The AD patients were composed of 39 women and 13 men, while the CG included 33 women and 15 men [(*χ*^2^ = 0.484 (1); *p* = 0.487)]. The mean age for the AD group was 84.40 (SD = 4.67), with a mean years of education of 6.36 (SD = 3.18), while the mean age for the CG was 82.54 (SD = 7.27), with a mean years of education of 7.15 (SD = 3.00). There were no significant differences between the two groups concerning age [*t*(98) = −1.536; *p* = 0.128)] or education [*t*(98) = 1.262; *p* = 0.210)]. All participants wrote a consent to be included in the study, which was approved by the Ethical Committee of Università Cattolica del Sacro Cuore di Milano.

### 2.2. Executive Functions Assessment

To obtain a thorough picture of the executive functioning of our sample, the following tests were administered to all participants: the Tower of London [43] to specifically evaluate planning abilities and The Trail Making Test, in its two versions, A and B [44]. Specifically, TMT-A evaluates attentional abilities, whereas TMT-B measures cognitive flexibility as components of executive functioning.

### 2.3. Virtual Reality Procedure

**Figure 1:**
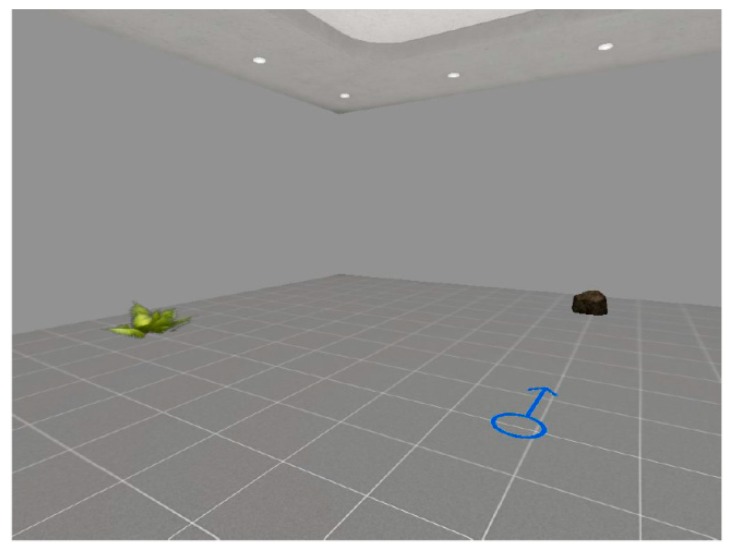
The VR-based procedure (encoding phase). In this phase, participants entered in the virtual room and they were asked to memorize the position of the plant.

A Virtual Reality (VR)—based procedure was used to evaluate the ability to retrieve and sync between different spatial reference frames [39]. First of all, participants were presented to VR technology to familiarize with it (i.e., approximately two–five minutes). In this phase, they entered a stimulus-free virtual room, and they were instructed on how to navigate inside it. Then, the VR-based procedure started, which consisted of two phases: a encoding and a retrieval phase. They entered in a virtual room which included two objects (i.e., a plant and a stone) and a blue arrow drawn on the floor, which pointed to the North (encoding phase) and signaled to participants the center of the testing room (see Figure 1).

Participants were invited to memorize the position of the plant, which was posited at the western part of the virtual room. The stone was located on the north side of the environment. Successfully, they were asked to retrieve its position in two different retrieval tasks (retrieval phase). The first task involved an aerial map of the room (i.e., a task that measures the ability to store an allocentric reference frame—Allocentric Abilities); the second task will involve entering the virtual room, but this time from another starting point (i.e., a task that measures the Mental Frame Syncing abilities [12,13,14]—Syncing Abilities). The accuracy of spatial location was the dependent variable in both retrieval tasks: 0 = poor answer (for example, choosing the same side of the retrieval point, i.e., the North); 1 = correct answer. This VR-based procedure was developed using NeuroVirtual 3D, a recent extension of the software NeuroVR (version 2.0, Milan, Italy) [45,46]. NeuroVirtual 3D software (http://www.neurovirtual.eu) provides a free virtual-reality platform for easily customizing virtual environments from a predefined library of existing ones (park, supermarket, station, etc.) that can be used for neuroscience research. It is composed of two modules: an Editor, for the customization of virtual scenes, and a Player, for the visualization of customized scenes in immersive (with the Vuzix Head Mounted Display) and not-immersive modality.

### 2.4. Procedure

Participants gave their informed consent to be included in the study. Then, they underwent the neuropsychological assessment to obtain a complete evaluation of their executive functioning. After the neuropsychological evaluation, participants were asked to perform the VR-based task. Participants were invited to sit comfortably in a quiet room in front of a portable computer (ACER ASPIRE with CPU Intel^®^ Core™i5 and graphic processor Nvidia GeForce GT 540M, 1024 × 768 resolution). A gamepad was used to explore and interact with the virtual room (Logitech Rumble F510). The training phase was delivered to allow participants interacting autonomously with VR (approximately two–five minutes). Then, the VR-based task started; its scope was to measure in two different retrieval tasks (i.e., “Allocentric Abilities” and “Syncing Abilities”) the ability to store and sync between different spatial reference frame.

### 2.5. Data Analyses

Preliminary, group comparisons among the different neuropsychological tests were carried by univariate analysis of covariance (ANCOVA), using age and education as covariates. As concerns behavioral indices from the VR-based tasks, group comparisons were examined by *χ*^2^ tests. Then, generalized linear models [47] were applied to investigate the contribution of the abilities in retrieving and syncing between spatial references frames (“Allocentric Abilities” or “Syncing Abilities”) to the executive functioning. These analyses included scores on MMSE as covariate predictors. Statistical analyses were performed in SPSS Statistics 21 (IBM, Armonk, NY, USA).

## 3. Results

### Group Differences Executive Functions and Spatial Reference Frames

Table 1 offers a picture of all data obtained from the neuropsychological assessment divided between the two groups and the statistical comparisons. When controlling for age and education, comparison of scores between groups using ANCOVA revealed significant differences as concerns all the tests considered (all *p* < 0.05). Patients suffering from AD performed significantly worse than healthy elderly individuals in all traditional tests evaluating executive functions (see Table 1). Results obtained from the *χ*^2^ tests indicated a significant difference among the AD and healthy elderly also regarding indices from the VR-based tasks (see Table 1). In particular, it emerged a significant difference among the two groups in the Allocentric Abilities, while just a trend to significance was observed in the Syncing Abilities (*p* = 0.06).

In Table 2 and Table 3, we reported results obtained from the generalized linear models to investigate the contribution of the Allocentric and Syncing Abilities (“Factor”) to executive functions (“Response”). In all models, scores on MMSE were inserted as covariates predictor.

On one hand, statistical analyses indicated a significant effect of the Allocentric Abilities on the performance in the Tower of London (*p* = 0.049, see Table 2). On the other hand, the Syncing Abilities on the first task of the Trail-Making Test, the TMT-A, had significant impact. In all models, the scores on MMSE significantly influenced the performance in tasks evaluating executive functions. No significant interaction effect was observed between abilities in using spatial reference frames (neither in “Allocentric Abilities” or “Syncing Abilities”) and scores on MMSE on executive functions. A trend to significance can be observed concerning the interaction “Syncing Abilities × MMSE” on TMT-A (*p* = 0.060).

## 4. Discussion

A growing body of evidence suggested that a decline in effectively using spatial reference frames for categorizing information occurs both in normal and pathological aging [15,48]. On the other hand, evidence indicated that: (1) an early decline in allocentric abilities is one of the first hallmarks of Alzheimer’s Disease (AD) [15]; (2) executive deficits primarily characterize the cognitive profile of older individuals (the so-called “frontal lobe hypothesis” [49,50]), because of the anatomical and functional deterioration occurring in frontal lobes. Literature underlined an association between the ability to use spatial reference frames and executive functions [29,30,31]. 

In this direction, the objective of the current study was to understand the contribution of abilities in using spatial reference frames in executive functioning. Indeed, findings obtained up to now raise the question: to what extent can spatial reference frames impairments have an influence on executive functions in both healthy and pathological aging?

First of all, our results confirmed but also extended previous knowledge about cognitive deficits early manifested in AD [15,16,51,52,53,54,55,56,57]. Our outcomes pointed out the presence of executive deficits among individuals with AD. In particular, they manifested difficulties in different aspects of executive functioning, from planning (Tower of London), to attentional and multitasking abilities (Trail Making Test A and B).

In literature, there were contrasting results about the executive impairments in the first phase of AD [54,55]. For instance, Collette and co-workers highlighted that patients with AD reported executive impairments related to controlled attention and working memory. This implies the involvement of parietal-temporal cortex regions that are importantly affected in AD. In fact, the anatomical correlates of executive impairments in the disease are still under debate, but an early disconnection in the parietal-temporal network has been proposed [58].

Understanding which are the cognitive deficits early affected in AD is of primary importance; our results highlighting the presence of large executive impairments in the first stage of this disease could be of value for the early detection of subtle cognitive weaknesses [59].

In the same perspective, our findings highlighted the presence of spatial reference frames impairments among individuals with AD, consistent with previous studies in this field [15]. The cognitive profile of AD appears to be characterized by an early decline in allocentric retrieval, combined with an early decline in other subtle neurocognitive mechanisms useful to support the allocentric-to-egocentric switching, i.e., the Mental Frame Syncing [12,13,60], linked to brain changes occurring in hippocampal regions [60,61,62,63] and in retrosplenial cortex [64,65,66].

Beyond the important possibility of early detection of cognitive impairments among individuals in the first stage of AD, another crucial research area that deserves attention is that related to the relationship between these early cognitive impairments manifested by patients.

In particular, as concerns the contribution of abilities in using spatial reference frames to executive functioning, an interesting pattern emerged from our results. Indeed, our findings indicated that allocentric functions contributed significantly to planning abilities, while syncing abilities influenced significantly attentional ones. It is possible to explain these results considering that allocentric elaborations require an active cognitive elaboration of the external environment, relying on the continuous construction of relations between spatial representations and landmarks, while the ability in syncing between different spatial reference frames recruits more attentional resources (see also [67,68,69,70]).

The efforts in understanding the relationship between cognitive weaknesses in AD population may also open interesting rehabilitation possibilities. For example, Serino and colleagues found that a VR-based training specifically built for the empowerment of the “Mental Frame Syncing” in a sample of patients with AD led also to an improvement in some tests tapping executive functioning (i.e., Verbal Fluency Test, Verbal Categorical Test, and FAB) [37]. Influencing higher-order cognitive abilities, such as the executive functions, the spatial reference frames appear to dramatically impact the way individuals categorize information. 

Eventually, from our study, the role of VR emerged as an advanced neuroscientific tool for assessing in ecological way complex spatial functions [71,72], also with pathological populations (for an example see [73]). Future studies should further exploit the potential of VR both to set-up innovative instruments able to detect early deficits or to support training in AD population, adopting also more immersive solutions [74,75], and to connect spatial reference frames to internal bodily states [76] within the “embodied medicine” perspective [77].

However, there are some limitations in our study that we should consider. The sample we recruited for this study was rather small, although well-matched for the main sociodemographic characteristics. Second, it would have been crucial to include in the study a group of patients suffering from MCI, mainly to evaluate the association between abilities in using spatial reference frames and executive functioning also in this intermediate stage of pathological aging to deeply investigate the role of executive functions and spatial reference frames as diagnostic markers of AD. Moreover, we could not deeply investigate the neural mechanisms involved in executive functions or the association with other cognitive abilities, such as the episodic memory [53], which is the another crucial cognitive marker of AD.

## Figures and Tables

**Table 1:** Scores obtained from executive functions and spatial references for healthy elderly individuals and patients suffering from Alzheimer’s Disease.

	Healthy Elderly Group	AD Group	F	*p*	Partial *ηp* ^²^
Tower of London (ToL) ^1^	23.13 ^2^ (7.76)	18.38 ^2^ (8.37)	10.678	0.002	0.100
Trail Making Test—TMT-A ^1^	101.64 ^2^ (70.23)	236.94 ^2^ (182.31)	17.865	<0.001	0.164
Trail Making Test—TMT-B ^1^	261.01 ^2^ (195.19)	530.27 ^2^ (272.55)	11.704	0.001	0.122
Allocentric Abilities	89.6% ^3^	71.2% ^3^	5.298 ^4^	0.021	0.23 ^5^
Syncing Abilities	35.4% ^3^	19.2% ^3^	3.318 ^4^	0.060	0.181 ^5^

^1^ For Tower of London (TOL), higher scores meant better performance; for Trial Making Test (TMT-A and TMT-B), higher scores meant worst performance. ^2^ Data are shown as means and standard deviations (SD). ^3^ Percentage of correct response; ^4^ *χ*^2^ test; ^5^ Effect size for χ^2^ test: $\varphi \;=\;\sqrt{\frac{\chi^{2}}{n}}$.

**Table 2:** Association between allocentric abilities and executive functions.

	B	Standard Error	*χ* ^2^	*p*
Tower of London				
Allocentric Abilities	21.507	109.160	3.882	0.049
MMSE	0.942	0.2276	17.127	<0.001
Allocentric Abilities * MMSE	−0.788	0.4576	0.2966	0.085
Trail Making Test—TMT-A				
Allocentric Abilities	10.079	1,852.216	0.003	0.957
MMSE	−16.716	3.919	18.240	<0.001
Allocentric Abilities * MMSE	1.338	77.624	0.030	0.863
Trail Making Test—TMT-B				
Allocentric Abilities	419.823	4,405.849	0.908	0.341
MMSE	−22.863	95.138	5.775	0.016
Allocentric Abilities * MMSE	−11.997	184.083	0.425	0.515

* Interaction between variables.

**Table 3:** Association between syncing abilities and executive functions.

	B	Standard Error	*χ* ^2^	*p*
Tower of London				
Syncing Abilities	77.392	107.488	0.518	0.47
MMSE	0.798	0.3455	5.341	0.021
Syncing Abilities * MMSE	−0.246	0.4145	0.352	0.553
Trail MakingTest—TMT-A				
Syncing Abilities	−378.637	1,845.662	−16.894	0.040
MMSE	−27.474	60.083	20.909	<0.001
Syncing Abilities * MMSE	13.354	71.118	3.526	0.060
Trail Making Test—TMT-B				
Syncing Abilities	−572.617	4,549.136	1.584	0.208
MMSE	−45.045	146.802	9.415	0.002
Syncing Abilities * MMSE	20.693	174.524	1.406	0.236

* Interaction between variables.
